# Sugarcane–Peanut Intercropping System Enhances Bacteria Abundance, Diversity, and Sugarcane Parameters in Rhizospheric and Bulk Soils

**DOI:** 10.3389/fmicb.2021.815129

**Published:** 2022-02-17

**Authors:** Ziqin Pang, Nyumah Fallah, Peiying Weng, Yongmei Zhou, Xiumei Tang, Muhammad Tayyab, Yueming Liu, Qiang Liu, Yijie Xiao, Chaohua Hu, Yongjun Kan, Wenxiong Lin, Zhaonian Yuan

**Affiliations:** ^1^Key Laboratory of Sugarcane Biology and Genetic Breeding, Ministry of Agriculture, Fujian Agriculture and Forestry University, Fuzhou, China; ^2^College of Agricultural, Fujian Agriculture and Forestry University, Fuzhou, China; ^3^Fujian Provincial Key Laboratory of Agro-Ecological Processing and Safety Monitoring, College of Life Sciences, Fujian Agriculture and Forestry University, Fuzhou, China; ^4^Key Laboratory of Crop Ecology and Molecular Physiology, Fujian Agriculture and Forestry University, Fuzhou, China; ^5^Cash Crops Research Institute, Guangxi Academy of Agricultural Sciences, Nanning, China; ^6^Province and Ministry Co-sponsored Collaborative Innovation Center of Sugar Industry, Guangxi University, Nanning, China

**Keywords:** sugarcane, intercropping, endosphere, rhizosphere and non-rhizosphere soil, bacterial diversity and abundance

## Abstract

Sugarcane–legume intercropping systems can effectively control pests and diseases as well as improve the fertility and health of farmland soil. However, little is known about the response of bacterial abundance, diversity, and community composition in the rhizosphere and non-rhizosphere soils under the sugarcane–peanut farming system. A field experiment was conducted with two treatments: sugarcane monoculture and sugarcane–peanut intercropping to examine the response of sugarcane parameters and edaphic factors. We also deciphered bacterial abundance, diversity, and community composition in the root endosphere, rhizosphere, and bulk soil by leveraging Illumina sequencing to conduct the molecular characterization of the 16S rRNA gene and nitrogenase (*nifH*) gene. We observed that sugarcane–peanut intercropping exhibited the advantages of tremendously increasing cane stalk height, stalk weight, and millable stalk number/20 m, and edaphic factors, namely, pH (1.13 and 1.93), and available phosphorus exhibited a fourfold and sixfold increase (4.66 and 6.56), particularly in the rhizosphere and bulk soils, respectively. Our result also showed that the sugarcane–peanut intercropping system significantly increased the bacterial richness of the 16S rRNA gene sequencing data by 13.80 and 9.28% in the bulk soil and rhizosphere soil relative to those in the monocropping sugarcane system, respectively. At the same time, sugarcane intercropping with peanuts significantly increased the Shannon diversity of nitrogen-fixing bacteria in the sugarcane rhizosphere soil. Moreover, most edaphic factors exhibited a positive regularity effect on bacterial community composition under the intercropping system. A linear discriminant analysis with effect size analysis of the 16S rRNA sequencing data revealed that bacteria in the root endosphere of the intercropped cane proliferated profoundly, primarily occupied by *Devosia*, *Rhizobiales*, *Myxococcales*, *Allorhizobium-Neorhizobium-Pararhizobium-Rhizobium*, *Bradyrhizobium*, and *Sphingomonas*. In conclusion, our findings demonstrated that sugarcane–peanut intercropping can enhance edaphic factors, sugarcane parameters, and bacterial abundance and diversity without causing adverse impacts on crop production and soil.

## Introduction

Sugarcane (*Saccharum officinarum* L.) is the world’s most crucial sugar and energy crop (Lu et al., 2021; Tayyab et al., 2021), and it is mainly cultivated in tropical and subtropical climates with an annual production of about 16 million tons worldwide (Chandel et al., 2012; Khalil et al., 2018). However, studies have established that cane growth and development are known to absorb a significant amount of soil nutrients because it produces large amounts of biomass (Oliveira et al., 2018; Camargo and Keeping, 2021). Therefore, it is of essence to adopt an ameliorative agricultural approach that can supply adequate nutrients to maintain the high productivity in the sugarcane plant cycle and minimize reduction in the following cycles. Among various farming practices, intercropping farming systems have been deemed as one of the sustainable agricultural practices globally (Tang et al., 2021). In particular, sugarcane–legumes intercropping systems have gained increasing traction in China and parts of Africa and have shown promising results in terms of production output, limiting N leaching (Himmelstein et al., 2016; Yin et al., 2018). Moreover, sugarcane–peanut uses natural resources effectively and efficiently; improves yields; minimizes the utilization of insecticides, pesticides, and chemical fertilizers (Wang et al., 2014; Solanki et al., 2019); reduces pests and disease outbreaks, and is also cost efficient (Chang et al., 2020). Sugarcane–legume intercropping systems can stimulate the proliferation of N-fixation by the legume’s bacteria, further promoting soil health and fertility and the overall environmental conditions, thereby mutually benefiting both plants (Solanki et al., 2020). Tang et al. (2021) demonstrated that sugarcane–peanut intercropping significantly improved soil phosphorus (P), available N, and available organic matter (OM) by 20.1, 65.3, and 56.0% in the root-soil relative to those in monocropping treatments. Similarly, Solanki et al. (2019) reported that sugarcane–legume intercropping systems profoundly increased soil-available potassium (K), total P, and the soil enzyme dehydrogenase. Moreover, numerous studies have shown that soil microbes in the rhizosphere and non-rhizosphere zones are responsive to intercropping systems (Howard et al., 2020; Liu et al., 2021).

Soil microecological environments are crucial in the growth and development of intercropped crops. Intercropping systems can promote soil microbial diversity and community composition, and soil biochemical properties in the rhizosphere (Cao et al., 2017; Tang et al., 2020) and non-rhizosphere soil (Wang et al., 2015), which may, in turn, contribute to soil ecosystem environments’ improvement (Zhou et al., 2019). A recent study established that intercropping farming systems induced changes in endophytic microbial community composition. Mei et al. (2021) report also mentioned that the maize-*Sonchus asper* intercropping farming pattern triggered a profound shift in endophytic bacterial community composition relative to those in *S. asper* monoculture. Additionally, intercropping with leguminous crops such as peanuts and soybeans can proliferate the activation of N-fixation through a process called biological nitrogen fixation (BNF) (Fallah et al., 2021). BNF has been deemed one of the potential systems to ameliorate the effect of N fertilizers and improve soil mineralization (Beneduzi et al., 2013) and sustainably boost crop productivity (Solanki et al., 2020). The symbiotically related diazotrophs and free-living could offer immense benefits in terms of colonization efficiency, thus enhancing efficiency through mutual interaction between the rhizosphere root zones and crops (Castro-González et al., 2011). Few studies have demonstrated the significance of sugarcane–legume-related N-fixation bacteria. For instance, Wang et al. (2014) and Solanki et al. (2019) revealed that the sugarcane–legume intercropping system enriched the beneficial N-fixers in the sugarcane–legume endosphere and rhizosphere soils, and these bacteria were classified as a plant growth-promoter. However, the existence of endophytic and symbiotically associated nitrogen-fixing bacteria in different compartments of soil such as bulk soil, rhizosphere soil, as well as tbe plant root endosphere of a non-leguminous crop such as cane and cane–peanut intercropping still need to be explored. With these issues in mind, a field experiment was established consisting of two treatments: sugarcane monoculture and sugarcane–peanut intercropping to investigate the response of sugarcane parameters and edaphic factors and decipher bacterial abundance, diversity, and community composition in the root endosphere, and rhizosphere and bulk soil by adopting Illumina sequencing to conduct the molecular characterization of the 16S rRNA gene and nitrogenase (*nifH*) gene. We hypothesized that: (a) sugarcane–peanut intercropping can increase the soil microorganisms’ abundance and diversity in sugarcane rhizosphere and bulk soil; (b) sugarcane–peanut intercropping affects the community composition of soil bacteria and nitrogen-fixing bacteria in the bulk soil, rhizosphere soil, and endosphere of sugarcane; and (c) edaphic factors can have significant impact on bacterial community composition.

## Materials and Methods

### Experimental Site, Design, and Treatments

**FIGURE 1:**
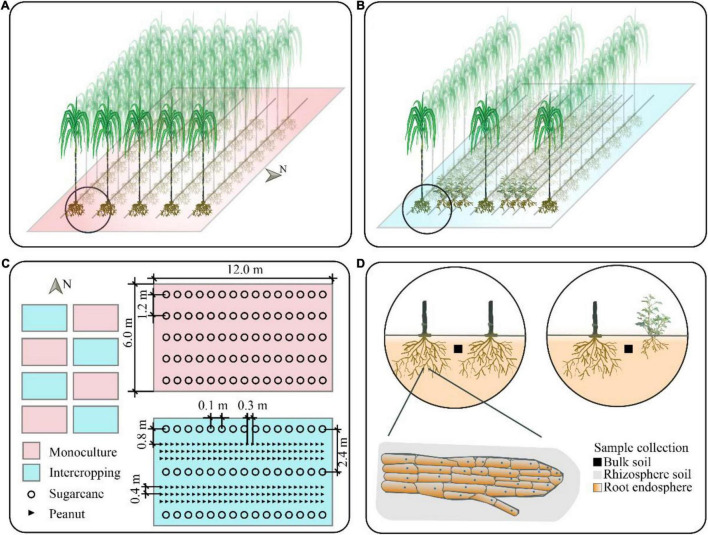
A diagram of the sugarcane monocropping and sugarcane–peanut intercropping field **(A–C)** and different compartments **(D)**; N stands for north direction.

The sugarcane–peanut intercropping field experiment was established at the Wuxuan Demonstration Base (23°56′64′′N, 109°51′26′′E), Luxin Town, Laibin City, Guangxi Province, China. Luxin Town is located in the south of the Tropic of Cancer, with a subtropical climate, and an annual average sunshine duration of 1,849.9 h and an annual average temperature of 21.2°C. The frost-free period was 330 days, and the annual average rainfall was 1,300 mm. Sugarcane variety (Liucheng 05-136) and the peanut (Guihua 1026) were obtained from the Cash Crops Research Institute of the Guangxi Academy of Agricultural Sciences. The site was previously used for sugarcane monoculture cropping system using a conventional approach. The following basic soil properties were measured: OM = 23.15 g/kg, total nitrogen (TN) = 1.17 g/kg, total phosphorus (TP) = 0.49 g/kg, and total potassium (TK) = 9.82 g/kg. The experiment was set in a randomized block design with two treatments and four replicates constituting a total of eight plots, with each covering an area of 72.0 m^2^ (12.0 × 6.0 m). The treatments included (i) sugarcane monoculture and (ii) sugarcane–peanut intercropping systems. Sugarcane and peanuts were simultaneously cultivated on March 15, 2018, after the soil was plowed (40 cm depth) using rotary tillage. Sugarcane monoculture was cultivated with a line spacing of 1.2 and 0.1 m row spacing. In the sugarcane monoculture field, each sett contained one bud, and a planting density of 83,333 buds/hm^2^. Sugarcane-peanut intercropped field had three lines of peanut planted adjacent to the sugarcane line. The distance between the sugarcane line was 2.4 m, with 0.1 m row spacing, and the distance between sugarcane and peanut was 0.8 m, while peanut consisted of 0.4 m row spacing and 0.3 m plant spacing. In the sugarcane–peanut plot, each sett (Fallah et al., 2021) contained two buds, and consisted of the same planting density in the sugarcane monoculture plot (Figure 1). The field was amended with calcium-magnesium-phosphate fertilizer (150 kg/ha) and mixed with silicon fertilizer (300 kg/ha), (N-P_2_O_5_-K_2_O 20-10-15). The peanut was supplemented with calcium-magnesium phosphate fertilizer (600 kg/ha). Two days after sowing, (1.2–1.5 L of 81.5% acetochlor per square hectometer) was diluted with 600–750 L of water and applied.

### Sampling and Preparation of Root, Bulk Soil, and Rhizosphere for DNA Extraction

On December 27, 2019, soil samples were collected, followed by sugarcane harvest. A shovel was used to dig sugarcane plants (≈0.5 m in height). The rhizosphere soil was detached from the roots of each plant; the loosely attached soil was gently collected by manually shaking the plant roots. A mono-rhi sample was soil collected from sugarcane root systems in a sugarcane monoculture field, whereas mono-bulk was soil collected outside the rhizosphere zone between the two rows of sugarcane. Accordingly, the rhizosphere soil collected from the root zone of plant in the sugarcane–peanut field was defined as int_rhi, while soil collected outside the rhizosphere zone between the two rows of sugarcane–peanut was defined as int_bulk. Each sample consisted of four replicates, and the soil of the same replicate was mixed; visible roots, straw and stones were removed from the samples, and the composite samples were homogenized and separated into two portions. Whirl-Pak^®^ bags were used to store the samples (Liu et al., 2021). Finally, a total of 24 samples were obtained and immediately transported to the laboratory, where it was stored at −20°C for DNA extraction and the determination of soil environmental variables. Plant roots were washed and surface-sterilized and frozen before DNA was extracted. Later, roots were washed in ethanol (100%) for 1 min, bleach (2.5%) 1 min, fresh bleach (2.5%) 30 min, and ethanol (100%) 1 min. The roots were taken to the sterile Erlenmeyer flasks aseptically and rinsed using ddH_2_O. Finally, the rhizosphere, bulk soil and roots were stored at −20°C awaiting DNA extraction (Gagnon et al., 2020).

### Estimation of Sugarcane Parameters

Twenty cane plants were selected randomly in each row, and each plant’s diameter and height were calculated in centimeters (cm) using a tape and a Vernier. The total number of cane plants sampled on a 20-m line in a plot was defined as millable stalk number (20 m), whereas available stalk numbers were considered the total number of cane plants sampled in an entire plot (10^3^/hm^2^). Cane plants’ sucrose content was determined using the approach described by Jackson and Jayanthy (2014), whereas theoretical sugarcane production was measured using the equations below (Lin et al., 2013):

Single stalk weight (kg) = [stalk diameter (cm)]^2^ × [stalk height (cm) - 30] × 1 (g/cm^3^) × 0.7854/1000.

Theoretical production (kg/hm^2^) = single stalk weight (kg) × productive stem numbers (hm^2^).

### Measurement of Soil Properties

The bulk soil and rhizosphere soil samples were used to measure soil environmental variables. A Sartorius PB-10 (Germany) pH meter was adopted to estimate soil pH (1:2.5 soil/water suspensions) (Bi et al., 2010). Soil available phosphorus (AP) was measured using the molybdenum blue method (Scrimgeour, 2007). The alkaline hydrolyzable diffusion method was used to extract soil available potassium (AK) (Kwon et al., 2009). Soil OM was estimated using the Walkley−Black approach, which comprised the soil OM oxidation by H_2_SO_4_ and K_2_Cr_2_O_7_, and FeSO_4_ was then used for titration (Fu et al., 2015) whereas soil available nitrogen (AN) was estimated using the alkaline hydrolysis diffusion technique (Tang et al., 2021).

### DNA Extraction, PCR Amplification, Illumina MiSeq Sequencing, and Data Processing

The Fast DNATM Spin Kit (MP Biomedicals, LLC, Santa Ana, CA, United States) was adopted to extract total genomic DNA using 0.5 g fresh soil as the manufacturer’s instructions prescribed. The DNA quality and quantity were assessed by calculating their absorbance (A260 and 280 nm) using BioTek Synergy H1 Hybrid Multi-Mode Microplate Reader (BioTek, Agilent, Santa Clara, CA, United States). The hypervariable V3–V4 regions of the 16S rRNA gene were targeted using PCR primers 5′ CCTACGGGNBGCASCAG 3′ and 50 GACTACNVGGGTATCTAATCC 30 (Lundberg et al., 2013; Takahashi et al., 2014) for the characterization of bacterial communities of three replicates per sample on the Illumina MiSeq (Illumina Inc., San Diego, CA, United States) platform. Moreover, to avoid the amplification of mitochondrial RNA (mRNA) or plastidial RNA (pRNA) from eukaryotes, peptide nucleic acid (PNA) oligomers were added to the PCR mix (Lundberg et al., 2013). PCR amplification was performed with an initial denaturation at 94°C for 3 min, denaturation (5 cycles at 94°C) for 30 s, annealing at 45°C for 20 s, extension at 65°C for 30 s, denaturation (20 cycles at 94°C) for 20 s, annealing at 55°C for 20 s, extension at 72°C for 30 s, and a final extension at 72°C for 5 min. The resulting amplicons were used to carry out a next-generation sequencing library appropriate for sequencing on an Illumina MiSeq sequencer (Biomarker Technologies Corporation, Beijing, China).

FLASH was used to merge paired-end reads of the original DNA fragments (Tan et al., 2017), according to a sample-specific barcode assigned to each sample. Based on 97% similarity, all sequences were clustered at the same operational taxonomic unit (OTU). For each OTU, sequences were representatively selected to annotate the taxonomic information for every sequence using the Ribosomal Database Project (RDP) (Wang et al., 2007). Sequences with low quality were removed if they were not matched to the barcode and primer, sequences more than 200 nucleotides without ambiguous base pairs and high average quality score (Q ≥ 20). All sequences were clustered at 97% nucleotide similarity, thus resulting in 1806 OTUs for 16S rRNA bacteria. SILVA database (SILVA Release 138, Bacterial) was used for the taxonomic classification of the respective sequences of bacteria. Finally, the raw data were submitted to the NCBI Sequence Read Archive (accession no. PRJNA777300).

The amplification of *nifH* gene amplicon was conducted using the method adopted in our previous study (Fallah et al., 2021). In short, the N-fixation *nifH* gene was amplified with primer set PolF and PolR (Poly et al., 2001). Both Illumina adaptor sequences and barcode sequences were employed to modify the amplicon (Caporaso et al., 2012). Purified PCR products were used to obtain sample libraries. The Miseq 300 cycle Kit was used for paired-end sequencing on a Miseq benchtop sequencer (Illumina, San Diego, CA, United States). The separation of *nifH* gene was done based on their barcodes, permitting up to one mismatch. Later, quality trimming was carried out with the adoption of Btrim (Kong, 2011). The forward and reverse reads were then merged into full-length sequences using FLASH (Magoc and Salzberg, 2011). Sequences containing ambiguous or short bases were removed. FRAMEBOT program was used to analyze *nifH* gene sequences (Wang et al., 2013). Sequences containing frameshift errors were removed, and sequences without error were later translated into conceptual proteins sequences. Next, the DOTUR program was adopted to cluster *nifH* gene protein sequences into OTUs with a 0.05 sequence distance cutoff (Zhou et al., 2016). Samples were refined to 10,000 sequences for each sample, and singletons were discarded. *nifH* OTUs taxonomic assignment was done by searching representative sequences against reference *nifH* with identified taxonomic information.

In the 16S rRNA sequencing data, 1,889,025 pairs of reads were obtained by sequencing 24 samples, and a total of 1,802,492 clean tags were generated after splicing and filtering double-ended reads. At least 44,717 clean tags were generated for each sample, and an average of 75,104 Clean tags was generated. The average GC content of 16S bacterial rRNA was 56.08%, with bases containing a quality value greater than or equal to 30, accounting for 90% of the total number of bases. After subsampling each sample to an equal sequencing depth (22,713 reads per sample) and clustering, 1,806 OTUs at 97% identity were obtained, with the number of OTUs ranging from 854 to 1,400 per sample (Supplementary Table 1). For *nifH* sequencing data, 1,827,259 pairs of reads were obtained by sequencing 24 samples, and a total of 1,652,439 clean tags were generated after splicing and filtering of double-ended Reads. At least 43,200 clean tags were generated for each sample, and an average of 68,852 clean tags was generated. The DNA of N-fixation bacteria contained an average GC content of 58.98%, and the percentage of bases with a quality value greater than or equal to 30, with the total number of bases greater than 97.5%. After subsampling each sample to an equal sequencing depth (29,753 reads per sample) and clustering, 1,320 OTUs at 97% identity were obtained, with the number of OTUs ranging from 180 to 633 per sample (www.biocloud.net) (Supplmementary Table 2).

### Statistical Analysis

Quantitative Insight into Microbial Ecology and R software (version3.6.1) (R Core Team, 2014) were employed to investigate endophytic and N-fixation bacterial communities’ richness (ACE) (Chao and Lee, 1992) and diversity (Shannon) (Keylock, 2005). Principle coordinate analysis (PCoA) with Bray–Curtis distance was adopted to explore and to visualize similarities or dissimilarities of bacterial community composition under both cropping systems and various soil compartments. The analysis of similarities (ANOSIM) was employed to further test significant differences between bacterial community composition in both cropping systems and the soil compartments. Redundancy analysis (RDA) was adopted to separately examine the correlations between soil environmental parameters and bacterial community composition obtained from *nifH* genes and 16S rRNA gene sequencing. Mantel tests were also adopted to calculate the relationship between the soil ecological variables and bacterial community composition for *nifH* genes and 16S rRNA gene sequencing data using the “vegan” package. Pearson’s correlation coefficients were employed to test the relationship between sugarcane parameters and bacteria in root endosphere, rhizosphere soil, and bulk soil. The differential abundance analysis of soil bacteria was conducted by employing the R package DESeq2 (Love et al., 2016). Volcano plot analysis was then conducted using R language-based package ggtern and grid, an extension of package ggplot2 to assess enriched genera among the different groups of samples for each sequencing data. Later, bacterial community compositions overlap and unique enriched genera were visualized using Venn diagrams^1^ in each sequencing data. Differences in the relative abundance of bacterial OTUs were assessed using the LEfSe (linear discriminant analysis with effect size) tool^2^. LefSe identified the statistical significance, effect size, and biologic consistency by classifying differentially abundant bacteria taxa (Segata et al., 2011). The indicators between the two groups of different farming modes, such as sugarcane yield trait indicators, were analyzed using Student’s *t*-test, while the indicators between the multi groups, such as soil nutrients, were compared using the LSD method for differential analysis.

## Results

### Effect of Intercropping on Sugarcane Growth

The different farming modes profoundly influenced sugarcane agronomic traits. Sugarcane stalk height and weight proliferated significantly in sugarcane–peanut intercropping by 7.60 and 22.03%, respectively, relative to those in the monocropping sugarcane system. Additionally, sugarcane’s millable stalk number sampled in a 20 m line in a plot increased significantly under the sugarcane–peanut intercropping system compared with the monocropping sugarcane system, but the available stalk number in the entire plot (10^3^/hm^2^) revealed a reverse trend. It was also observed that the sugarcane stem diameter, sucrose content, and yield results showed no difference in the two modes of crop cultivation (Table 1).

**TABLE 1:** Sugarcane agronomic parameters under Sugarcane-peanut intercropping and monocropping sugarcane.

	Stalk height (cm)	Stalk diameter (cm)	Sucrose content (%)	Single stalk weight (kg)	Millable stalk number (20 m)	Available stalk number(10^3^/hm^2^)	Production (10^3^kg/hm^2^)
Sugarcane monocropping	250.1 ± 5.3 b	2.62 ± 0.02 a	14.65 ± 0.39 a	1.18 ± 0.02 b	171 ± 4 b	71.1 ± 1.5 a	84.2 ± 0.5 a
Sugarcane–peanut intercropping	269.1 ± 9.2 a	2.77 ± 0.07 a	14.77 ± 0.19 a	1.44 ± 0.04 a	271 ± 5 a	56.5 ± 1.0 b	81.3 ± 3.8 a

*Different lowercase letters indicate significant difference at P < 0.05.*

### Effect of Intercropping on Rhizospheric and Bulk Soil Properties

**FIGURE 2:**
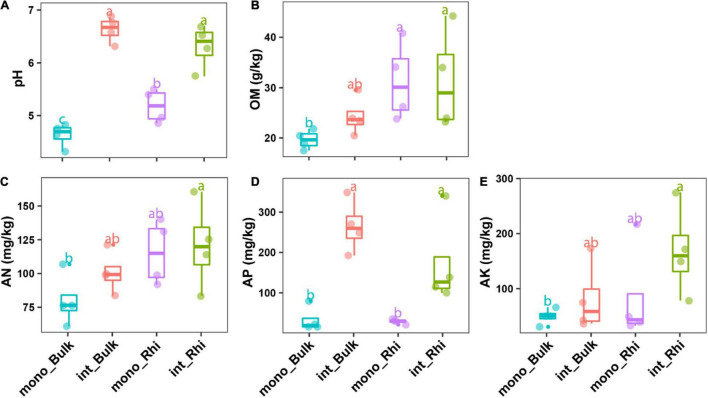
Sugarcane rhizosphere and non-rhizosphere edaphic factors under sugarcane intercropping and monocropping modes. Boxes with different lowercase letters indicate significant differences between various regimes based on the LSD test (*p* < 0.05). **(A)** pH, **(B)** organic matter (OM), **(C)** available nitrogen (AN), **(D)** available phosphorus (AP), and **(E)** available potassium (AK).

Soil edaphic factors differed remarkably between the two cultivation systems, with soil pH and available phosphorus (AP) showing a significant difference (*p* < 0.001) in the entire samples (Figures 2A,D). However, soil OM, AN, and AK revealed no significant difference between the two cultivation systems (Figures 2B,C,E). Regarding the variation of edaphic soil factors in the rhizosphere and non-rhizosphere soil under the two cultivation systems, it was shown that soil pH values in the rhizosphere and non-rhizosphere soil increased by 1.13 and 1.93; while soil AP exhibited a fourfold and sixfold increase (4.66 and 6.56) under sugarcane–peanut cultivation compared to sugarcane monoculture, respectively (Figures 2A,D). On the contrary, OM, AN, and AK showed no significant difference in rhizosphere compared to non-rhizosphere soil under both cultivation systems (Figures 2B,C,E). Two-factor ANOVA revealing the effects of soil farming systems and different soil regions demonstrated that soil factors, namely, pH and AP were greatly influenced by the different farming systems, whereas OM and AN were considerably affected by the different soil regions. In addition, soil pH was significantly affected by the interaction between the farming systems and the various soil locations (Supplementary Table 5).

### Effect of Different Cropping Systems on Bacterial α Diversity in Rhizosphere and Non-rhizosphere Soil

**FIGURE 3:**
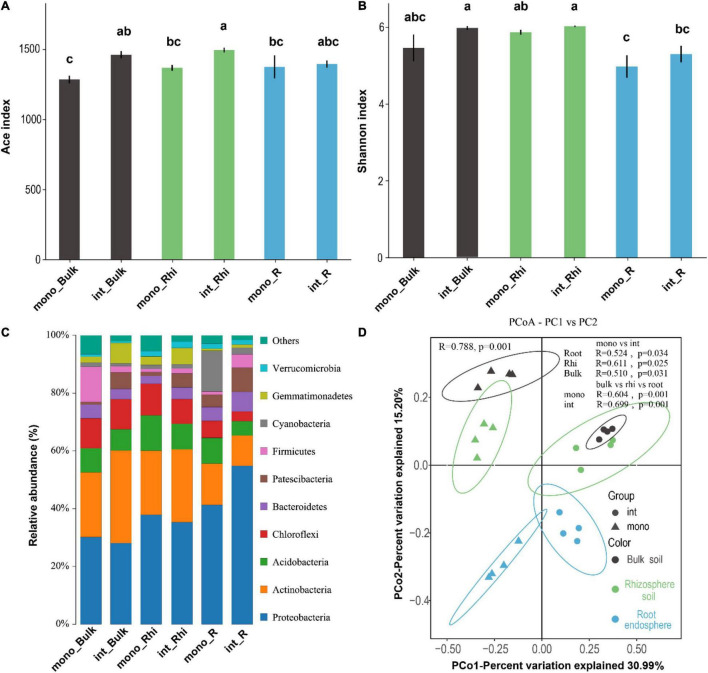
Bar graph depicting alpha diversity indices, including **(A)**, microbial community richness (ACE) and **(B)**, microbial community diversity (Shannon), bacterial relative abundance **(C)**. PCoA with Bray–Curtis distance showing similarities or dissimilarities of bacterial community composition under the both cropping systems and various soil compartments. ANOSIM indicating the significant difference between bacterial community composition in both cropping systems and the soil compartments **(D)**.

**FIGURE 4:**
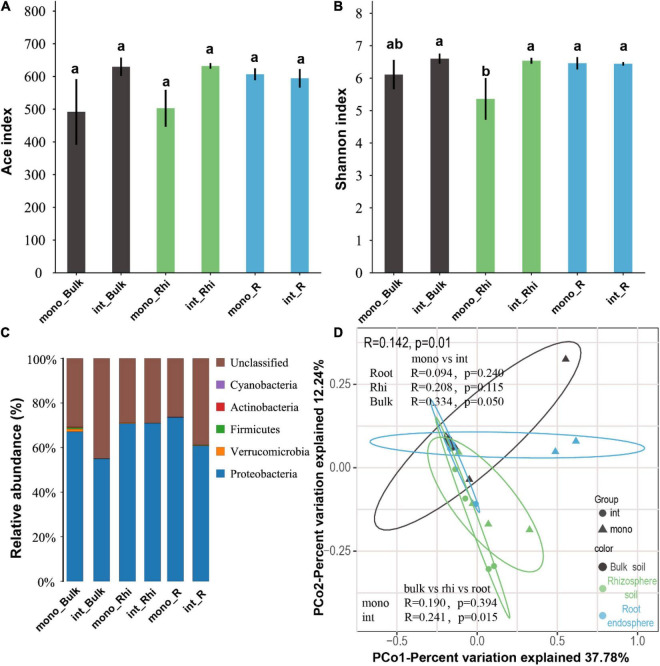
Ace index: nitrogen-fixing bacteria richness index **(A)**, Shannon index: nitrogen-fixing bacteria community diversity index **(B)**, nitrogen-fixing bacteria relative abundance **(C)** and nitrogen-fixing bacterial community composition. Principle coordinate analysis (PCoA) with Bray-Curtis distance showing similarities or dissimilarities of N-fixation bacterial community composition under the both cropping systems and various soil compartments. ANOSIM indicating the significant difference between N-fixation bacterial community composition in both cropping systems and the soil compartments **(D)**.

The coverage for the observed OTUs was 98.86 ± 0.03% (mean ± sem) and the rarefaction curves showed clear asymptotes (Supplementary Figure 1A), which together demonstrated a near-complete sampling of the community. The bacterial richness (ACE) and diversity (Shannon) indices were explored in the sugarcane rhizosphere zone, root, and bulk soil under sugarcane–peanut cultivation system and sugarcane monocropping. The result revealed that the bacterial ACE index increased by 13.80 and 9.28% (*p* < 0.05) in the rhizosphere soil and bulk soil under the sugarcane–peanut intercropping system relative to those in the sugarcane monocropping system, respectively (Figure 3A). We also observed that bacterial diversity did not significantly differ in the bulk and rhizosphere soil under both farming systems. Moreover, in the sugarcane root endosphere, bacterial diversity diminished considerably (*p* < 0.05) in both farming systems compared to those in the bulk and rhizosphere samples (Figure 3B). In both cropping systems, the amount bacteria identified were more, primarily driven by *Proteobacteria* (28.10–54.90%), *Actinobacteria* (10.50–32.00%), and *Acidobacteria* (4.90–12.20%) were the dominant soil bacteria identified in both farming systems, followed by *Chloroflexi* (3.40–11.00%), *Bacteroidetes* (2.70–6.80%), *Patescibacteria* (0.90–8.40%), *Firmicutes* (1.1–12.2%), Cyanobacteria (1.0–14.15%), *Gemmatimonadetes* (0.70–7.00%), and *Verrucomicrobia* (0.60–2.20%) (Figure 3C). Noticeably, *Proteobacteria* proliferated significantly (*p* < 0.05) in intercropping root zone compared to the sugarcane monocropping root system. Additionally, in sugarcane–peanut intercropping bulk soil, *Gemmatimonadetes* revealed significant improvement (*p* < 0.05) than the sugarcane monocropping system. However, it was also observed that *Firmicutes* and *Cyanobacteria* were significantly diminished (*p* < 0.05) in intercropping bulk soil and intercropping rhizosphere soil than those in the monoculture bulk soil and the monoculture rhizosphere soil, respectively (Supplementary Table 3). PCoA showed the overall similarity of the bacterial community structure between samples using the OTUs of 16S rRNA sequencing data. PCo1 represented 30.99%, while PCo2 accounted for 15.20% of the changes detected in bacterial community composition. We also observed that the different cultivation systems profoundly influenced bacterial community composition in the three compartments. Moreover, bacterial community composition in the root endosphere, bulk and rhizosphere soils were distinctly apart from one another (Figure 3D). Anosim further confirmed that the bacterial community composition was significantly influenced by the different farming systems compared with different soil locations (*p* ≤ 0.001). On the other hand, *nifH* community composition showed little difference in bulk soil of different farming systems, whereas in the intercropping system, the *nifH* community composition was significantly (*p* ≤ 0.05) affected in different soil locations (Figure 4D).

### Effect of Different Cropping Systems on N-Fixation Bacterial α Diversity in Rhizosphere and Non-rhizosphere Soil

The coverage for the observed OTUs was 99.75 ± 0.01% (mean ± SEM) and the rarefaction curves showed clear asymptotes (Supplementary Figure 1B). N-fixation community ACE and Shannon indices in the rhizosphere zone, root system, and bulk soil under the sugarcane–peanut cultivation system and sugarcane monocropping were also tested. We observed that N-fixation bacterial community richness revealed no significant difference in the entire samples under both monocropping and intercropping systems (Figure 4A). In contrast, N-fixation bacterial community diversity showed a considerable improvement in intercropping rhizosphere soil relative to those in the sugarcane monocropping system (Figure 4B). A little number of N-fixation bacteria were identified in both cropping systems. Phyla *Proteobacteria* (54.98–73.51%), *Firmicutes* (0.02–0.65%), *Verrucomicrobia* (0.00–1.21%), and *Cyanobacteria* (0.00–0.16%) were the dominant N-fixation bacteria detected in the various samples under the two treatments (Figure 4C). However, no significant variation among the bacteria in the different samples was observed (Supplementary Table 4). PCo1 represented 37.78%, whereas PCo2 accounted for 12.24% of the N-fixation bacteria community composition changes. The analysis also demonstrated N-fixation bacteria community composition was clustered together in the entire samples under the different cultivation systems (Figure 4D).

### Association Between Bacterial Community Composition and Soil and Plant Parameters

**FIGURE 5:**
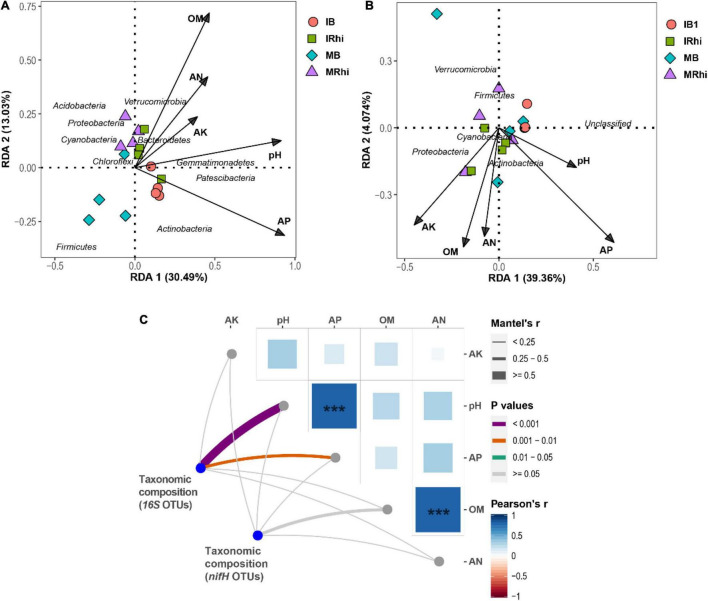
Redundancy analysis (RDA) of sequencing data of bacteria (16S rRNA) **(A)**, and N-fixation bacteria (*nifH*) and ecological parameters **(B)**. Pairwise comparisons of soil edaphic variables are shown with a color gradient representing Pearson’s correlation coefficients **(C)**. Taxonomic composition of bacteria (16S OTUs) and N-fixing bacteria (*nifH* OTUs) association with soil environmental variables, displayed by partial Mantel tests in the rhizospheric soil and non-rhizospheric soil under sugarcane–peanut farming pattern and sugarcane monocropping. The width of each edge matches with Mantel’s r statistic for the corresponding distance correlations. Note: asterisk mark denotes the significance level. ***p < 0.001.

Bacterial community compositions displayed a high association with intrinsic soil edaphic factors. The association between the essential soil edaphic factors and bacterial community compositions were discerned using RDA analysis. The results demonstrated significant variations in the bacterial community composition of the 16S rRNA data under both farming systems. Noticeably, Verrucomicrobia and Bacteroidetes demonstrated a strong and positive correlation with soil AK, OM, and AN, whereas Firmicutes revealed the opposite. Furthermore, Acidobacteria, Proteobacteria, and Cyanobacteria showed a significant and negative association with soil AP (Figure 5A). N-fixation bacteria, namely, Actinobacteria, demonstrated a significant and positive association with soil AN, whereas Proteobacteria and Cyanobacteria were significantly and positively connected with soil AK. However, Firmicutes and Verrucomicrobia were significantly and negatively related to soil AK, AN, and OM (Figure 5B). Mantel test analysis demonstrated that the taxonomic composition of bacteria (16S OTUs) exhibited significant solid correlations with soil pH and AN (Figure 5C). The Pearson’s correlation between the parameter of sugarcane and bacteria in the root endosphere, rhizosphere soil, and bulk soil demonstrated that most of the sugarcane growth parameters were significantly correlated with bacteria in the rhizosphere soil and root endosphere rather than the bulk soil. Noticeably, *Allorhizobium-Neorhizobium-Pararhizobium-Rhizobium* and *Pseudonocardia* in the sugarcane root showed considerable and positive association with the sugarcane weight and sucrose content, respectively, *Lysinibacillus* and *Psychrobacillus* in the sugarcane root exhibited a significant and positive association with the sugarcane stalk number, while *Solibacillus* in the rhizosphere soil was significantly and positively associated with the sugarcane diameter (Supplementary Table 6).

### Differentially Abundant Bacteria Under Different Cropping Systems

LEfSe analysis was adopted to detect differentially abundant bacteria (16S rRNA) in the bulk soil, rhizosphere soil, and root endosphere, comparing the two cultivation systems (Supplementary Figure 2). The analysis demonstrated that *Acidobacteriaes*, *Actinospicaceae*, *Frankiales*, and *Ktedonobacteraceae* were enriched in both the bulk soil and rhizosphere soil under the single cropping system compared to the intercropping system. In contrast, *Micromonosporaceae*, *Solirubrobacterales*, and *A4b* were more dominant in the bulk soil and rhizosphere soil under the intercropping system than the monoculture system. It was also observed that some bacteria proliferations were unique either in the bulk soil or rhizosphere soil. For instance, *Nocardioidaceae*, *Pseudonocardiaccae*, *Gemmatimonadaceae*, and *Dongiaceae* proliferation were unique in the bulk soil of intercropped plants, while *Saccharimonadales* and *Myxococcales* were dominant in the rhizosphere soil of the same farming systems (Supplementary Figures 2A,B). In the root endosphere of the sugarcane–peanut farming system, *Allorhizobium-Neorhizobium-Pararhizobium-Rhizobium* and *Devosia* enriched significantly, while *Actinospica* and *Dyella* were higher in the sugarcane monocropping root zone (Supplementary Figure 2C).

**FIGURE 6:**
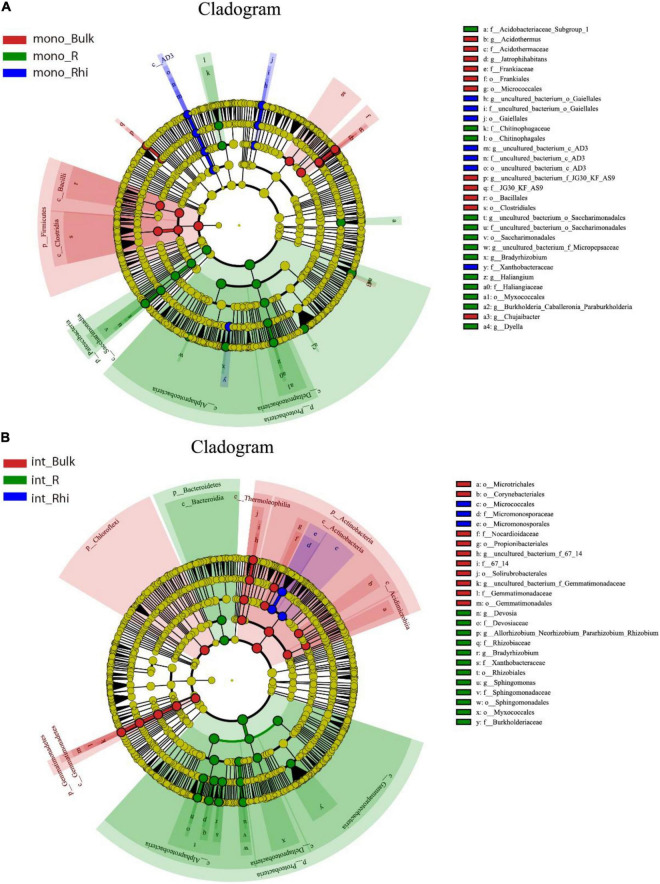
LEfSe analysis depicting the significant discriminant taxa (16S rRNA) among bulk soil, rhizosphere soil and root endosphere in **(A)** monoculture and **(B)** intercropping system (LDA score threshold: ≥ 4.0). Different colored regions represent different species. The circles from inside to out represent the classification levels from the phylum to the genus. Each small filled circle represents a classification at this level, and size is proportional to relative abundance. Bulk, bulk soil; Rhi, rhizosphere soil; R, root endosphere.

**FIGURE 7:**
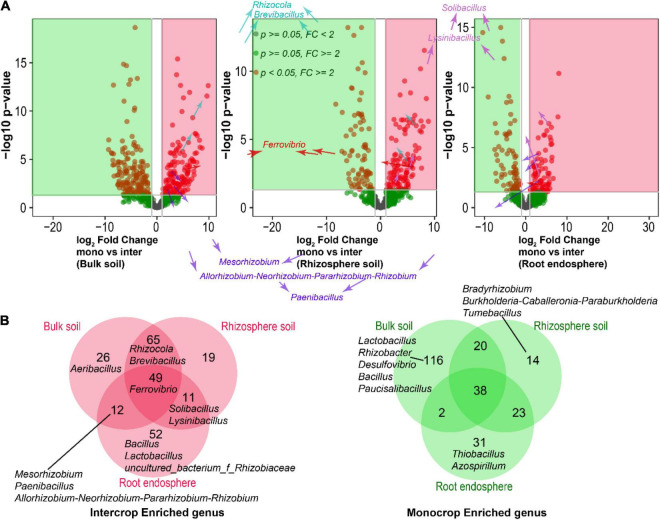
Volcano plots depicting enriched (green) and depleted (red) bacteria in mono vs. inter (bulk soil), mono vs. inter (rhizosphere soil) and mono vs. inter (root endosphere) **(A)**, followed by Venn diagram illustrating unique and overlap enriched genera (16S rRNA) under sugarcane–peanut intercropping (red) and sugarcane monocropping (green) **(B)**.

LEfSe analysis was also used to better understand the differentially abundant bacteria (16S rRNA) by comparing the three samples. The results showed that the bulk soil was characterized by *Acidothermus*, *Jatrophihabitans*, and *Chujaibacter* in monoculture, while Bradyrhizobium potentially occupied the rhizosphere soil. In the root endosphere, *Haliangium*, *Burkholderia-Caballeronia-Paraburkholderia*, and *Dyella* in monoculture increased profoundly (Figure 6A). In sugarcane–peanut intercropping, the bulk soil was primarily occupied by *Nocardioidaccac* and *Gemmatimonadaceae* relative to those in the rhizosphere soil and root endosphere. *Micromonosporaceae* was the dominant bacteria in the rhizosphere soil under the intercropping system. Interestingly, in the root endosphere of the intercropped plants, a significant number of endophytic bacteria genera were enriched, primarily occupied by *Devosia*, *Rhizobiales*, *Myxococcales*, *Allorhizobium-Neorhizobium-Pararhizobium-Rhizobium*, *Bradyrhizobium*, and *Sphingomonas* proliferated profoundly (Figure 6B). DESeq2 differential analysis was performed in each soil location to analyze the bacteria genera that were significantly enriched or depleted in the different farming systems (Figure 7A). The results showed that sugarcane–peanut intercropping enriched bacteria in the different soil compartments compared with the single-cropping system. For example, *Rhizocola* and *Brevibacillus* in the rhizosphere and bulk soils were significantly enriched under the sugarcane–peanut farming system. Whereas *Solibacillus* and *Lysinibacillus* were significantly improved in the rhizosphere soil and root endosphere of sugarcane–peanut intercropping. Moreover, *Mesorhizobium*, *paenibacillus* and *Allorhizobium-Neorhizobium-Pararhizobium-Rhizobium* in the bulk soil and root endosphere were significantly enriched under the sugarcane–peanut farming system, while *Ferrovibrio* was significantly improved in the bulk soil and roots (Figure 7A). Volcano plot analysis was then employed to assess the differential abundance of bacteria (16S rRNA) in the different groups (mB vs. iB, mRhi vs. iRhi and mR vs. iR). The analysis showed that *Mesorhizobium* and *Allorhizobium-Neorhizobium-Pararhizobium-Rhizobium* in the bulk soil and root endosphere were significantly improved in intercropping compared with monocropping. In the sugarcane monoculture system, some nitrogen-fixing bacteria were also enriched, namely, *Rhizobacter* and *Desulfovibrio* in bulk soil, followed by *Bradyrhizobium* and *Tumebacillus* in rhizosphere soil and *Azospirillum* in root endosphere (Figure 7A). We then used a Venn diagram analysis to unveil the unique and overlap genera (16S rRNA) in the compared groups. The analysis revealed that intercropping exhibited the advantage of enriching 49 bacteria genera, while 38 enriched genera were identified in the monocropping system of the entire samples (Figure 7B).

Diazotrophic differential abundance analysis showed that Azohydromonas, Azoarcus, Pelomonas, Azospirillum, Paraburkholderia, Azotobacter, Burkholderia, and *Acidiphilium* were more permanent under the monocropping samples compared to those under the intercropping system. In contrast, sugarcane–peanut intercropping was predominantly enriched by *Azotobacter*, *Geobacter*, *Acidiphilium*, *Azoarcus*, and *Azohydromonas* (Supplementary Figure 3A). LEfSe analysis was adopted to gain further insight into N-fixation differential abundance by comparing the three groups of samples in each farming system. The analysis further confirmed that more N-fixation differential abundance, including *Bradyrhizobium*, *Rhizobiales*, *Azospirillum*, and *Rhosdospirillales* in monoculture farming rhizosphere soil outperformed those in the bulk soil and root endosphere (Supplementary Figure 3B). Moreover, the overall N-fixation differential abundance in the entire samples under the sugarcane–peanut intercropping system significantly diminished (Supplementary Figure 3C).

## Discussion

Sugarcane–legumes cultivation systems are highly effective and efficient farming systems that have the potential to increase crop productivity in an eco-friendly manner (Chogatapur et al., 2017). Li Y. et al. (2013) reported that the dry weight of crops biomass and yield of sugarcane plants were profoundly increased by 35.44 and 30.57% under the sugarcane–soybean cultivation system. In a related study, Shen et al. (2018) revealed that sugarcane–peanut intercropping significantly increased the yield, pol, brix, sucrose, and purity of sugarcane. Correspondingly, it was observed that the stalk height, stalk weight, and millable stalk number increased tremendously, likely explained by the increase in soil nutrients, namely, pH and AP. This phenomenon could also be attributed to more effective utilization of light, land resources, and nutrients (Quan et al., 2013). We also observed that the available stalk number (10^3^/hm^2^) decreased in sugarcane–peanut intercropping compared with the sugarcane monocropping field, which contradicts the findings reported by Malviya et al. (2021) and Solanki et al. (2020). We believed that the higher amount of tillers in the sugarcane–peanut intercropping field led to the decrease in the available stalk number (10^3^/hm^2^) (Nadiger and Nadagouda, 2017). Numerous studies have demonstrated that edaphic factors are sensitive to intercropping farming systems (Wang et al., 2015; Tang et al., 2020). In the current study, the sugarcane–peanut intercropping system exhibited the advantages of increasing soil pH and AP, particularly in the rhizosphere and non-rhizosphere soil, where soil pH increased by 1.13 and 1.93, and soil AP by 4.66 and 6.56 times, respectively, which was evident in sugarcane growth parameters. This finding corroborated with the results reported by Shen et al. (2018), which revealed that soil AP increased by 26.7, 16.0, and 65.3%, and soil pH increased by 1.6 and 3.0% under intercropping systems compared to monocropping farming systems.

Soil microbial richness and diversity are responsive to conservative farming systems such as sugarcane–legumes farming systems (Solanki et al., 2020). Furthermore, sugarcane–legumes farming systems are conducive to maintaining the soil microorganisms’ richness and diversity (Liu et al., 2021) and can also inhibit harmful microorganisms that are likely to occur in a single cultivation mode and interfere with general annoyance (Cong et al., 2015). In the sugarcane–peanut intercropping system, bacterial richness detected in the 16S rRNA sequencing data increased by 13.80 and 9.28% in the rhizosphere soil and bulk soil relative to those in the sugarcane monocropping system, respectively. This finding is consistent with a previous study (Li and Wu, 2018), wherein microbial OTUs’ diversity increased under cucumber intercropped with seven different crops, namely, alfalfa, trifolium, wheat, rye, chrysanthemum, rape, and mustard. This result seems to suggest that the root exudates of various plants can promote soil microbial taxa. The microbial community in sugarcane–peanut may directly interact with the crop roots, thereby stimulating the crop roots to release nutrients and exudates (Canarini et al., 2019).

Interactions between microbes and sugarcane–legumes farming systems have widely been documented (Li X. et al., 2013). In the current study, we identified many bacteria in 16S rRNA sequencing data compared to *nifH* sequencing data, with Proteobacteria exhibiting distinct patterns in various soil samples under both treatments. However, Proteobacteria proliferated significantly in the intercropping root zone compared to the sugarcane monocropping root. Consistently, a greater relative abundance of Proteobacteria population has been identified in the rhizosphere soils of sugarcane–legume intercropping isolates than the monoculture isolates in the rhizosphere soils (Solanki et al., 2019), which has been acknowledged as N-fixation bacteria (Fallah et al., 2021). This result suggested that Proteobacteria plays a crucial role in N-fixation due to their direct interaction with the crop roots in the intercropping farming system.

Soil bacterial community compositions are responsive to soil environmental parameters (Lian et al., 2019). Further, intercropping systems have widely been reported to have a noticeable impact on bacterial community compositions (Sun et al., 2009; Tian et al., 2019). In the current study, most soil edaphic factors exhibited a regularity effect on bacterial community composition under the intercropping system, specifically in the rhizosphere soil and bulk soil. Verrucomicrobia and Bacteroidetes exhibited a strong and positive association with soil AK, OM, and AN, whereas Firmicutes demonstrated the opposite. Moreover, Acidobacteria, Proteobacteria, and Cyanobacteria showed a significant and negative association with soil AP. N-fixation bacteria, namely, Actinobacteria, demonstrated a significant and positive association with soil AN, whereas Proteobacteria and Cyanobacteria were significantly and positively connected with soil AK. However, Firmicutes and Verrucomicrobia were significantly and negatively correlated with soil AK, AN, and OM (Figure 5B). This result confirmed with the finding documented by Lian et al. (2019), wherein environmental properties, such as soil organic carbon (SOC), nitrate nitrogen, ammonium nitrogen (NH_4_^+^), nitrate (NO_3_^–^), dissolved organic carbon (DOC), and pH were the principal determinant impacting bacteria dissimilarities in rhizosphere soil under sugarcane–soybean intercropping.

LEfSe analysis revealed that a significant number of bacteria genera (16S rRNA) in the root endosphere of the intercropped cane proliferated profoundly, primarily occupied by *Devosia*, *Rhizobiales*, *Myxococcales*, *Allorhizobium-Neorhizobium-Pararhizobium-Rhizobium*, *Bradyrhizobium*, and *Sphingomonas* compared with sugarcane monocropping. It has been established that *Devosia* is known for its bioremediation potential, with a high presence in an environment contaminated with toxins. However, Honeker et al. (2019) detected a considerably higher amount of *Devosia* in the rhizosphere of buffalo grass than the bulk soil, which partly conforms with our finding. *Rhizobiales* bacteria are widely associated with plants’ growth and development by providing phytohormones, essential nutrients, and precursors vital for plants’ metabolites (Delmotte et al., 2009; Verginer et al., 2010). The high abundance of rhizosphere-competent *Rhizobiales* in the intercropping soil can be explained by the beneficial plant–soil feedback endured during evolutionary periods (Garrido-Oter et al., 2018). According to Wang et al. (2020), *Myxococcales* are well distributed in agricultural soil. Contrary to our finding, Dang et al. (2020) reported that *Myxococcales* diminished significantly under proso millet and mung bean intercropping systems. It has been reported that *Allorhizobium-Neorhizobium-Pararhizobium-Rhizobium* is a rhizobia N-fixer (You et al., 2021), and *Bradyrhizobium* is one of the genera of N-fixing bacteria in leguminous crops capable of forming symbiotic nodules, whereas *Sphingomonas* is widely regarded as the best environmentally friendly approach for phosphorus nutrition mobilization for plants (Zhang et al., 2018). Consistently, Lindström and Mousavi (2019) identified and described these bacteria as nitrogen-fixing bacteria of legumes.

## Conclusion

In summary, our results demonstrated that soil sugarcane–peanut intercropping systems exhibited the advantage of tremendously increasing the aboveground growth of sugarcane. Moreover, the sugarcane–peanut farming pattern showed a regulatory effect on the soil edaphic factors, namely, pH and AP, and the bacterial richness of the 16S rRNA sequencing was more pronounced in the rhizosphere soil bulk soil and root endosphere relative to those in the sugarcane monocropping system. We also observed that a significant number of bacteria genera of 16S rRNA sequencing in the root endosphere of the intercropped plants proliferated profoundly, including, *Devosia*, *Rhizobiales*, *Myxococcales*, *Allorhizobium-Neorhizobium-Pararhizobium-Rhizobium*, *Bradyrhizobium*, and *Sphingomonas*. Additionally, most of the soil edaphic factors demonstrated a strong and positive correlation with 16S rRNA bacterial community composition under the intercropping system, specifically in the rhizosphere and bulk soil. These results suggest that the sugarcane–peanut intercropping pattern could potentially impact soil nutrients, cane agronomic parameters, peanut yield, and bacteria in sugarcane root systems compared to the monoculture farming system.

## Data Availability Statement

The datasets presented in this study can be found in online repositories. The names of the repository/repositories and accession number(s) can be found in the article/Supplementary Material.

## Author Contributions

ZP, XT, and ZY designed the research. ZP, YX, and XT conducted the experiments. ZP, NF, and YZ analyzed the data. NF, PW, and ZP wrote the manuscript. YL, QL, MT, CH, YK, and WL reviewed the manuscript. ZY supervised the work and approved the manuscript for publication. All authors contributed to intellectual input and provided assistance for this study and for manuscript preparation.

## Conflict of Interest

The authors declare that the research was conducted in the absence of any commercial or financial relationships that could be construed as a potential conflict of interest.

## Publisher’s Note

All claims expressed in this article are solely those of the authors and do not necessarily represent those of their affiliated organizations, or those of the publisher, the editors and the reviewers. Any product that may be evaluated in this article, or claim that may be made by its manufacturer, is not guaranteed or endorsed by the publisher.

## Footnotes

1
http://bioinfogp.cnb.csic.es/tools/venny/index.html

2
www.biocloud.net
