# Supplementary material for: Sugarcane–Peanut Intercropping System Enhances Bacteria Abundance, Diversity, and Sugarcane Parameters in Rhizospheric and Bulk Soils
Source: Front Microbiol. 2022 Feb 17;12:815129. doi: 10.3389/fmicb.2021.815129 (PMC8891962; doi:10.3389/fmicb.2021.815129)
Supplement: Supplementary file 1 [file Data_Sheet_1.docx]

Supplementary Material

| **TABLE S1 \|** Bacterial 16S rRNA Sequencing Data Quality Assessment | | | | | | | | | | |
| --- | --- | --- | --- | --- | --- | --- | --- | --- | --- | --- |
| Sample ID | PE Reads | Clean Tags | Effective Tags | AvgLen(bp) | GC(%) | Q20(%) | Q30(%) | Effective(%) | OTU_Number | Coverage |
| mono_bulk1 | 80046 | 76229 | 72370 | 415 | 55.65 | 96.69 | 91 | 90.41 | 1230 | 0.9912 |
| mono_bulk2 | 80276 | 76582 | 72660 | 416 | 58.06 | 96.69 | 91 | 90.51 | 919 | 0.9869 |
| mono_bulk3 | 80014 | 77174 | 75170 | 413 | 57.71 | 97.01 | 91.63 | 93.95 | 1132 | 0.9904 |
| mono_bulk4 | 79681 | 74345 | 70106 | 417 | 54.43 | 96.05 | 89.67 | 87.98 | 1097 | 0.99 |
| mono_rhi1 | 80204 | 76344 | 71686 | 413 | 57.42 | 96.57 | 90.72 | 89.38 | 1242 | 0.9902 |
| mono_rhi2 | 80086 | 77034 | 71206 | 414 | 57.21 | 96.84 | 91.22 | 88.91 | 1263 | 0.9901 |
| mono_rhi3 | 79936 | 75597 | 71926 | 413 | 57.09 | 96.48 | 90.55 | 89.98 | 1162 | 0.99 |
| mono_rhi4 | 80300 | 77023 | 70556 | 413 | 56.72 | 96.76 | 91.13 | 87.87 | 1168 | 0.9896 |
| mono_r1 | 80193 | 77063 | 70930 | 413 | 55.96 | 96.92 | 91.38 | 88.45 | 1008 | 0.9859 |
| mono_r2 | 80329 | 77339 | 72365 | 415 | 55.43 | 96.72 | 90.96 | 90.09 | 1068 | 0.987 |
| mono_r3 | 79779 | 77024 | 72488 | 413 | 55.45 | 96.86 | 91.22 | 90.86 | 854 | 0.9866 |
| mono_r4 | 48581 | 44717 | 41778 | 415 | 55.57 | 95.1 | 87.67 | 86 | 1400 | 0.9871 |
| int_bulk1 | 80151 | 76340 | 67514 | 413 | 56.69 | 96.52 | 90.6 | 84.23 | 1341 | 0.9884 |
| int_bulk2 | 80095 | 76850 | 72604 | 415 | 56.4 | 96.79 | 91.05 | 90.65 | 1237 | 0.9891 |
| int_bulk3 | 79695 | 75498 | 71504 | 415 | 56.44 | 96.48 | 90.47 | 89.72 | 1272 | 0.9893 |
| int_bulk4 | 80141 | 76435 | 69140 | 413 | 56.82 | 96.68 | 90.91 | 86.27 | 1290 | 0.9882 |
| int_rhi1 | 80019 | 76459 | 72805 | 413 | 56.31 | 96.75 | 91.13 | 90.98 | 1317 | 0.9888 |
| int_rhi2 | 80184 | 76829 | 71970 | 415 | 56.21 | 96.62 | 90.86 | 89.76 | 1383 | 0.9897 |
| int_rhi3 | 79828 | 77139 | 72942 | 415 | 56.26 | 96.89 | 91.36 | 91.37 | 1266 | 0.989 |
| int_rhi4 | 79879 | 75511 | 72778 | 416 | 55.73 | 96.24 | 90.06 | 91.11 | 1366 | 0.9894 |
| int_r1 | 80137 | 76097 | 72690 | 413 | 54.58 | 96.4 | 90.38 | 90.71 | 1119 | 0.9879 |
| int_r2 | 80133 | 77162 | 74280 | 415 | 54.97 | 96.77 | 91.06 | 92.7 | 1247 | 0.9875 |
| int_r3 | 79671 | 75578 | 71550 | 413 | 54.08 | 96.37 | 90.33 | 89.81 | 1039 | 0.986 |
| int_r4 | 79667 | 76123 | 72208 | 416 | 54.65 | 96.56 | 90.7 | 90.64 | 1225 | 0.9881 |
| Sum/ave | 1889025 | 1802492 | 1695226 | 414 | 56.08 | 96.57 | 90.71 | 89.68 | 1806 | 0.9886 |

| **TABLE S2** **\|** *nifH* Gene Sequencing Data Quality Assessment | | | | | | | | | | |
| --- | --- | --- | --- | --- | --- | --- | --- | --- | --- | --- |
| Sample ID | PE Reads | Clean Tags | Effective Tags | AvgLen(bp) | GC(%) | Q20(%) | Q30(%) | Effective(%) | OTU_Num | Coverage |
| mono_bulk1 | 80252 | 76042 | 75750 | 318 | 62.17 | 98.98 | 97.7 | 94.39 | 180 | 0.9989 |
| mono_bulk2 | 80015 | 73877 | 70153 | 306 | 59.95 | 99.02 | 97.76 | 87.67 | 485 | 0.9975 |
| mono_bulk3 | 79830 | 74643 | 68472 | 304 | 58.1 | 98.99 | 97.7 | 85.77 | 568 | 0.9978 |
| mono_bulk4 | 80077 | 73918 | 66514 | 299 | 56.88 | 99.1 | 97.94 | 83.06 | 594 | 0.9979 |
| mono_rhi1 | 80231 | 71873 | 67564 | 307 | 59.9 | 98.95 | 97.61 | 84.21 | 587 | 0.9973 |
| mono_rhi2 | 70778 | 60773 | 56581 | 310 | 62.19 | 99.09 | 97.9 | 79.94 | 538 | 0.9963 |
| mono_rhi3 | 80073 | 73283 | 67488 | 302 | 58.03 | 98.97 | 97.66 | 84.28 | 530 | 0.9978 |
| mono_rhi4 | 80214 | 73804 | 66207 | 307 | 60.39 | 99.08 | 97.89 | 82.54 | 633 | 0.9964 |
| mono_r1 | 80059 | 71930 | 69667 | 313 | 62.6 | 98.99 | 97.7 | 87.02 | 312 | 0.9984 |
| mono_r2 | 80123 | 75169 | 72584 | 312 | 62.65 | 99.04 | 97.82 | 90.59 | 391 | 0.9972 |
| mono_r3 | 79821 | 74061 | 66996 | 301 | 57.89 | 99.05 | 97.81 | 83.93 | 566 | 0.9975 |
| mono_r4 | 79942 | 72392 | 66503 | 299 | 56.06 | 99.06 | 97.87 | 83.19 | 573 | 0.9977 |
| int_bulk1 | 53185 | 43200 | 40190 | 304 | 57.41 | 98.91 | 97.54 | 75.57 | 592 | 0.9976 |
| int_bulk2 | 58058 | 48066 | 44341 | 300 | 56.46 | 99.07 | 97.89 | 76.37 | 602 | 0.9977 |
| int_bulk3 | 67867 | 57919 | 53920 | 302 | 56.85 | 98.91 | 97.56 | 79.45 | 579 | 0.9978 |
| int_bulk4 | 56863 | 46813 | 42850 | 303 | 56.47 | 99.09 | 97.92 | 75.36 | 624 | 0.9974 |
| int_rhi1 | 79956 | 70182 | 66885 | 307 | 61.86 | 99.01 | 97.72 | 83.65 | 531 | 0.9973 |
| int_rhi2 | 80006 | 73459 | 68058 | 310 | 62.07 | 99.03 | 97.76 | 85.07 | 530 | 0.9968 |
| int_rhi3 | 79751 | 75020 | 68830 | 303 | 59.39 | 99 | 97.71 | 86.31 | 596 | 0.9969 |
| int_rhi4 | 79942 | 75166 | 71171 | 306 | 59.42 | 99.04 | 97.81 | 89.03 | 540 | 0.9971 |
| int_r1 | 79887 | 73897 | 68958 | 300 | 56.3 | 98.95 | 97.64 | 86.32 | 549 | 0.9975 |
| int_r2 | 80111 | 72087 | 69258 | 308 | 59.06 | 99.09 | 97.9 | 86.45 | 470 | 0.9973 |
| int_r3 | 80077 | 73938 | 68452 | 301 | 56.79 | 98.97 | 97.68 | 85.48 | 577 | 0.9976 |
| int_r4 | 80141 | 70927 | 65516 | 301 | 56.53 | 99.07 | 97.9 | 81.75 | 632 | 0.9977 |
| Sum/ave | 1827259 | 1652439 | 1542908 | 305 | 58.98 | 99.02 | 97.77 | 84.06 | 1320 | 0.9975 |

**TABLE S3 |** Bacterial Abundance Identified in the 16S rRNA Sequencing Data in The Rhizosphere and Non-rhizosphere Soils under Both Farming Systems.

| Phylum | mono_Bulk | int_Bulk | mono_Rhi | int_Rhi | mono_R | int_R |
| --- | --- | --- | --- | --- | --- | --- |
| Proteobacteria | 30.18cd | 28.10d | 37.90bc | 35.36bcd | 41.30b | 54.87a |
| Actinobacteria | 22.39abc | 32.03a | 22.19abc | 25.18ab | 14.31bc | 10.48c |
| Acidobacteria | 8.31ab | 7.35ab | 12.22a | 8.85ab | 8.91ab | 4.94b |
| Chloroflexi | 10.41a | 10.44a | 10.95a | 8.55ab | 5.92ab | 3.35b |
| Bacteroidetes | 4.75a | 3.55a | 2.70a | 4.07a | 4.78a | 6.80a |
| Patescibacteria | 0.91b | 5.72ab | 1.35b | 4.78ab | 4.26ab | 8.35a |
| Firmicutes | 12.20a | 2.14b | 1.10b | 1.73b | 1.06b | 4.69ab |
| Cyanobacteria | 1.48b | 1.03b | 1.38b | 1.45b | 14.15a | 2.25b |
| Gemmatimonadetes | 2.00c | 7.01a | 2.91bc | 5.67ab | 0.72c | 1.06c |
| Verrucomicrobia | 0.78a | 0.57a | 1.91a | 2.23a | 1.71a | 1.71a |
| Others | 6.58a | 2.05b | 5.39a | 2.12b | 2.88b | 1.50b |

Values followed by different lowercase letters with in the same row are significantly different at the 0.05 robability level.

**TABLE S4 |** N-fixing Bacterial Community Abundance under Sugarcane-Peanut Intercropping and Sugarcane Monocropping

| Phylum | mono_Bulk | int_Bulk | mono_Rhi | int_Rhi | mono_R | int_R |
| --- | --- | --- | --- | --- | --- | --- |
| Actinobacteria | 0.34a | 0.00a | 0.00a | 0.00a | 0.05a | 0.00a |
| Cyanobacteria | 0.00a | 0.00a | 0.00a | 0.00a | 0.16a | 0.00a |
| Firmicutes | 0.65a | 0.09ab | 0.04ab | 0.08ab | 0.02b | 0.12ab |
| Proteobacteria | 67.16ab | 54.98b | 70.81a | 70.92a | 73.51a | 60.8ab |
| Unclassified | 30.63ab | 44.91a | 28.76b | 28.96ab | 26.25b | 38.8ab |
| Verrucomicrobia | 1.21a | 0.02a | 0.39a | 0.04a | 0.00a | 0.28a |

Values followed by different lowercase letters with in the same row are significantly different at the 0.05 probability level.

| **Table S5** \| Two-factor analysis of variance between the two farming systems and soil location of soil edaphic factors. | | | |
| --- | --- | --- | --- |
|  | Farming mode (Fm) | Soil region (Sr) | Fm*Sr |
| pH | 103.093*** | NS | 7.907*** |
| OM | NS | 7.819*** | NS |
| AN | NS | 5.628*** | NS |
| AP | 31.89*** | NS | NS |
| AK | NS | NS | NS |

| **Table S6** \| The Pearson's correlation between sugarcane parameters and bacteria in root endosphere, rhizosphere soil and bulk soil. | | | | |
| --- | --- | --- | --- | --- |
| Parameter of sugarcane | Genus | Region | R value | p value |
| weight | Roseiarcus | Bulk soil | -0.95 | 0.05 |
| weight | SWB02 | Bulk soil | 0.99 | 0 |
| weight | Variovorax | Bulk soil | -0.95 | 0.05 |
| weight | Dyadobacter | Root | 0.95 | 0.05 |
| weight | Goodfellowiella | Root | -0.96 | 0.05 |
| weight | Pajaroellobacter | Root | -0.98 | 0.02 |
| weight | Rhodoplanes | Root | 0.95 | 0.05 |
| weight | Allorhizobium-Neorhizobium-Pararhizobium-Rhizobium | Root | 0.95 | 0.05 |
| weight | Altererythrobacter | Root | 0.95 | 0.05 |
| weight | uncultured_bacterium | Root | -0.98 | 0.02 |
| weight | uncultured_bacterium_f_Chitinophagaceae | Root | -0.95 | 0.05 |
| weight | Ktedonobacter | Rhi | -0.96 | 0.05 |
| weight | Actinospica | Rhi | -0.98 | 0.02 |
| weight | Occallatibacter | Rhi | -0.95 | 0.05 |
| weight | Psychrobacillus | Rhi | 0.99 | 0.01 |
| weight | SWB02 | Rhi | 0.95 | 0.05 |
| weight | Thermosporothrix | Rhi | -0.98 | 0.02 |
| weight | uncultured_bacterium_f_Acidobacteriaceae_Subgroup_1 | Rhi | -0.96 | 0.05 |
| weight | uncultured_bacterium_f_BIrii41 | Rhi | 0.95 | 0.05 |
| weight | uncultured_bacterium_f_Magnetospiraceae | Rhi | 0.97 | 0.03 |
| weight | uncultured_bacterium_f_Sandaracinaceae | Rhi | 0.95 | 0.05 |
| weight | uncultured_bacterium_f_bacterium_endosymbiont_of_Onthophagus_Taurus | Rhi | 0.98 | 0.02 |
| weight | uncultured_bacterium_o_R7C24 | Rhi | 1 | 0 |
| weight | uncultured_bacterium_o_S085 | Rhi | 0.98 | 0.02 |
| weight | Bradyrhizobium | Rhi | -0.98 | 0.02 |
| weight | Brevibacillus | Rhi | 0.96 | 0.05 |
| weight | Catenulispora | Rhi | -0.95 | 0.05 |
| Sucrose | uncultured_bacterium_f_Sporichthyaceae | Bulk soil | 0.95 | 0.05 |
| Sucrose | Pseudonocardia | Root | 0.95 | 0.05 |
| Sucrose | uncultured_bacterium_f_Microscillaceae | Root | 1 | 0 |
| Sucrose | uncultured_bacterium_f_candidate_division_TM7_bacterium_JGI_0001002-L20 | Root | 0.95 | 0.05 |
| production | Amycolatopsis | Rhi | -0.96 | 0.05 |
| stalk number(m) | Thermoflavimicrobium | Bulk soil | 0.95 | 0.05 |
| stalk number(m) | uncultured_bacterium_f_BIrii41 | Bulk soil | 0.98 | 0.02 |
| stalk number(m) | uncultured_bacterium_f_Roseiflexaceae | Bulk soil | 0.95 | 0.05 |
| stalk number(m) | Lysinibacillus | Root | 0.96 | 0.05 |
| stalk number(m) | Psychrobacillus | Root | 0.99 | 0.01 |
| stalk number(m) | Sinomonas | Root | -0.99 | 0 |
| stalk number(m) | Candidatus_Koribacter | Root | -0.99 | 0 |
| stalk number(m) | IS-44 | Rhi | 0.97 | 0.03 |
| stalk number(m) | Lapillicoccus | Rhi | -0.95 | 0.05 |
| stalk number(m) | Monashia | Rhi | -0.95 | 0.05 |
| stalk number(m) | Aquicella | Rhi | 0.95 | 0.05 |
| stalk number(m) | uncultured_bacterium_f_Rhodanobacteraceae | Rhi | 0.98 | 0.02 |
| stalk number(m) | Candidatus_Koribacter | Rhi | -0.99 | 0 |
| stalk number(m) | Candidatus_Udaeobacter | Rhi | -0.99 | 0 |
| stalk number(hm^2^) | Kribbella | Bulk soil | -0.96 | 0.05 |
| stalk number(hm^2^) | Dokdonella | Root | -0.95 | 0.05 |
| stalk number(hm^2^) | uncultured_bacterium_f_BIrii41 | Root | -0.98 | 0.02 |
| stalk number(hm^2^) | uncultured_bacterium_f_Ilumatobacteraceae | Root | -0.95 | 0.05 |
| stalk number(hm^2^) | Actinocorallia | Rhi | -0.95 | 0.05 |
| stalk number(hm^2^) | Microbacterium | Rhi | -0.95 | 0.05 |
| stalk number(hm^2^) | Allorhizobium-Neorhizobium-Pararhizobium-Rhizobium | Rhi | -0.98 | 0.02 |
| stalk number(hm^2^) | uncultured_bacterium_f_Sporichthyaceae | Rhi | -0.95 | 0.05 |
| stalk number(hm^2^) | Bosea | Rhi | -0.97 | 0.03 |
| stalk number(hm^2^) | Candidatus_Alysiosphaera | Rhi | -0.96 | 0.05 |
| height | Devosia | Bulk soil | 0.95 | 0.05 |
| height | Thiobacillus | Bulk soil | 0.95 | 0.05 |
| height | uncultured_bacterium_o_Obscuribacterales | Root | -0.95 | 0.05 |
| diamter | Faecalibacterium | Bulk soil | -0.96 | 0.05 |
| diamter | Phascolarctobacterium | Bulk soil | -0.96 | 0.05 |
| diamter | Thermoactinomyces | Bulk soil | 0.96 | 0.05 |
| diamter | uncultured_bacterium_o_S085 | Bulk soil | 0.95 | 0.05 |
| diamter | uncultured_bacterium_o_Acidobacteriales | Root | -0.98 | 0.02 |
| diamter | Roseiarcus | Rhi | -0.97 | 0.03 |
| diamter | Solibacillus | Rhi | 0.95 | 0.05 |
| diamter | uncultured_bacterium_f_bacterium_endosymbiont_of_Onthophagus_Taurus | Rhi | 0.95 | 0.05 |
| diamter | uncultured_bacterium_o_Acidobacteriales | Rhi | -0.95 | 0.05 |


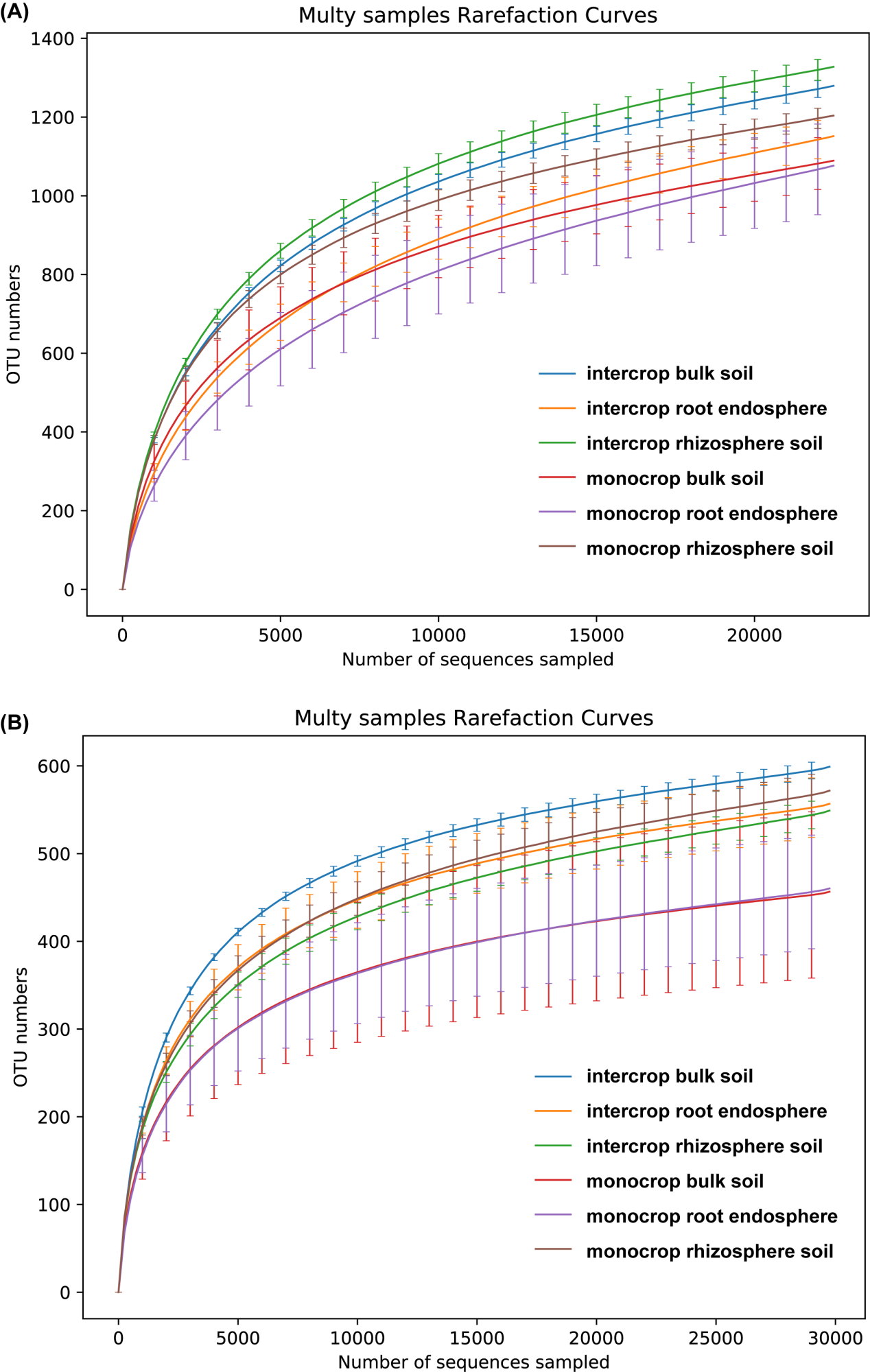
 **FIGURE S1** **|** The species accumulation curves displaying the adequacy for the sample size of bacteria (16S rRNA) (A) and N-fixation bacteria (B) under sugarcane-peanut intercropping and sugarcane monoculture.


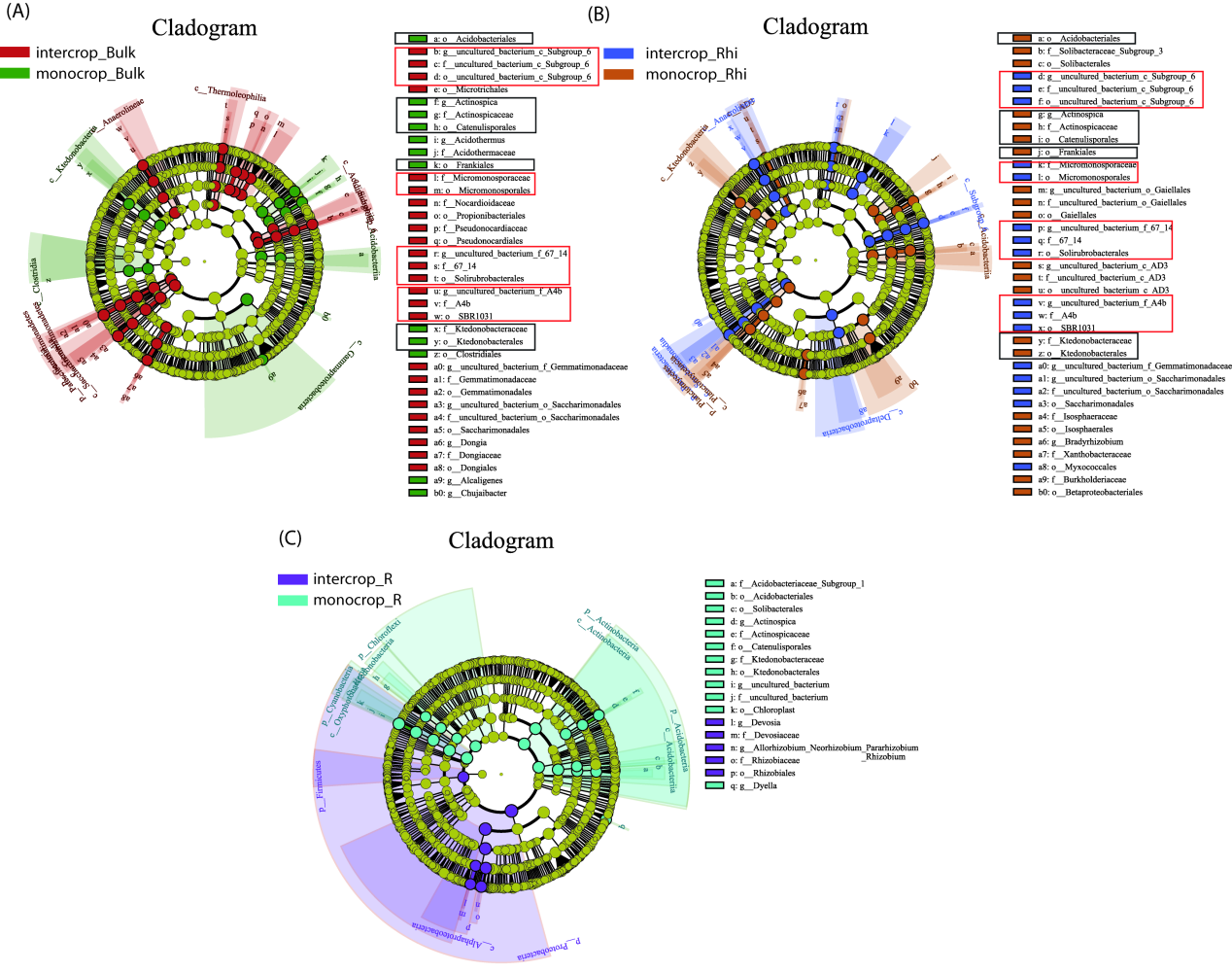


**FIGURE S2** **|** LEfSe depicts the significant discriminant taxa (16S rRNA) between monocropping sugarcane and intercropping sugarcane in **(A),** bulk soil, **(B)** rhizosphere soil and **(C)**, root endosphere species (LDA score threshold: ≥4.0). Different colored regions represent different species. The circles from inside to out represent the classification levels from the phylum to the genus. Each small filled circle represents a classification at this level, and size is proportional to relative abundance. Bulk, bulk soil; Rhi, rhizosphere soil; R, root endosphere. The box indicates that bulk soil and rhizosphere soil have a common enrichment of flora under sugarcane monocropping (black box), sugarcane peanut intercropping (red box).


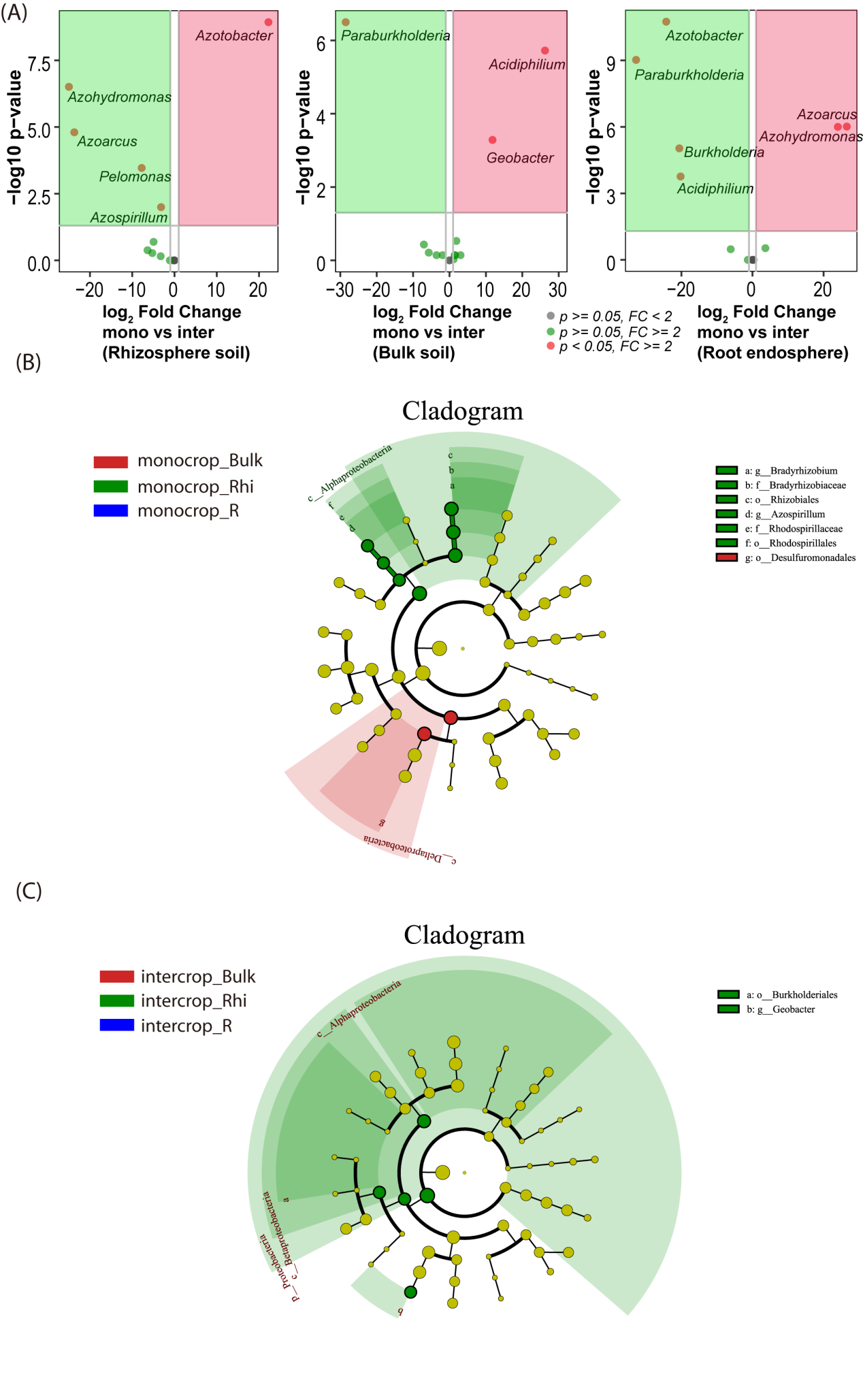


**FIGURE S3** **|** **(A)** Volcano plots depicting nitrogen fixing bacteria enriched (blue) and depleted (red) under mono vs inter (bulk soil), mono vs inter (rhizosphere soil) and mono vs inter (root endosphere). LEfSe analysis were used to determine the significant discriminant taxa among bulk soil, rhizosphere soil and root endosphere in **(B)** monoculture and **(C)** intercropping system (LDA score threshold: ≥2.0). Different colored regions represent different species. The circles from inside to out represent the classification levels from the phylum to genus. Each small filled circle represents a classification at this level, and size is proportional to relative abundance. Bulk, bulk soil; Rhi, rhizosphere soil; R, root endosphere.
